# Epidemiology of Curable Sexually Transmitted Infections among Women at Increased Risk for HIV in Northwestern Tanzania: Inadequacy of Syndromic Management

**DOI:** 10.1371/journal.pone.0101221

**Published:** 2014-07-15

**Authors:** Suzanna C. Francis, Trong T. Ao, Fiona M. Vanobberghen, Joseph Chilongani, Ramadhan Hashim, Aura Andreasen, Deborah Watson-Jones, John Changalucha, Saidi Kapiga, Richard J. Hayes

**Affiliations:** 1 MRC Tropical Epidemiology Group, London School of Hygiene and Tropical Medicine, London, United Kingdom; 2 Mwanza Intervention Trials Unit, National Institute for Medical Research, Mwanza, Tanzania; 3 National Institute for Medical Research, Mwanza, Tanzania; 4 Clinical Research Department, London School of Hygiene and Tropical Medicine, London, United Kingdom; International AIDS Vaccine Initiative, United States of America

## Abstract

**Background:**

Curable, non-viral pathogens account for a significant burden of sexually transmitted infections (STIs), and there is established evidence that STIs increase both HIV acquisition and transmission. We investigated the prevalence, trends, and factors associated with *Chlamydia trachomatis, Neisseria gonorrhoeae, Trichomonas vaginalis* and *Treponema pallidum*, and the performance of syndromic management, among a cohort of women working in bars, hotels, and other food and recreational facilities near large-scale mines in northwestern Tanzania.

**Methods:**

HIV-negative women aged 18–44 years (N = 966) were enrolled and followed for 12 months in a microbicides feasibility study. We collected sociodemographic and behavioural data, performed clinical examinations, and tested for STIs, at enrolment and 3-monthly. Risk factors for STIs were investigated using logistic regression models with random effects. Sensitivity, specificity and predictive values of syndromic management were calculated.

**Results:**

At enrolment, the prevalences of *C. trachomatis*, *N. gonorrhoeae*, *T. vaginalis*, and high-titre active syphilis were 111/956 (12%), 42/955 (4%), 184/945 (19%) and 46/965 (5%), respectively. There were significant decreases over time for *C. trachomatis* and *T. vaginalis* (OR trend per month: 0.94 [95% CI 0.91, 0.97]; and 0.95 [0.93, 0.98], respectively; both p<0.001). The majority of these infections were not diagnosed by the corresponding syndrome; therefore, most participants were not treated at the diagnosis visit. Syndromic management was poorly predictive of laboratory-diagnosed infections. We identified a number of risk factors for STIs, including low educational level, some sexual behaviours, and ever having been pregnant.

**Conclusions:**

This analysis demonstrates that the prevalences of curable STIs are high among women who work in food and recreational facilities in northwestern Tanzania. Most of these infections are missed by syndromic management. Accurate and affordable rapid-point-of-care tests and innovative interventions are needed to reduce the burden of STIs in this population which is at increased risk for HIV.

## Introduction

Despite steady advances in diagnosis, treatment, and public health interventions, sexually transmitted infections (STIs) continue to affect a large proportion of those living in resource-limited settings, particularly women of reproductive age [1]. Curable, non-viral pathogens (including *Chlamydia trachomatis*, *Neisseria gonorrhoeae*, *Trichomonas vaginalis* and *Treponema pallidum*) account for a significant burden of STIs [2]. There is established evidence that STIs increase both HIV acquisition and transmission [3]–[5]. Furthermore, untreated infections can lead to adverse reproductive health outcomes including pelvic inflammatory disease, infertility, premature labour, low birth weight, and congenital infections [6]–[8].

Women working in bars, hotels and other food and recreational facilities in Tanzania have been documented to have higher risk of STIs, including HIV, than the general population [9]–[12]. Studies in similar populations in Mbeya, Moshi, Mwanza city, and Mwanza region, Tanzania, reported prevalences of 6–13% for *C. trachomatis*, 4–22% for *N. gonorrhoeae*, 12–24% for *T. vaginalis*, and 8–24% for active infection with *T. pallidum* (i.e. active syphilis) [9], [10], [12], [13]. The Tanzanian Ministry of Health advocates syndromic management of STIs [14]. Syndromic management is based on the identification of a syndrome (a group of symptoms and easily recognised signs), and the provision of treatment targeting the organisms mainly responsible for causing the syndrome [15]. However, several recent papers have reported relatively poor performance of this approach among women due to most infections being asymptomatic [16], [17]. Better knowledge of the epidemiology of STIs among women who are most vulnerable to these infections may lead to improved treatment policies and prevention programmes to reduce their burden and mitigate subsequent reproductive health problems.

In this report, we aim to describe the prevalence, trends and risk factors of curable, non-viral STIs among women at increased risk for HIV infection in northwestern Tanzania who participated in a 12 month prospective cohort study designed to assess feasibility for conducting future microbicide trials. The background HIV prevalence in this population is 16%, and data on viral infections including HIV-1 and *Herpes simplex virus 2* (HSV-2) have been reported elsewhere [18].

## Methods

### Ethical statement

The study was approved by the Ethics Committees of the National Institute for Medical Research and London School of Hygiene and Tropical Medicine. All participants gave written informed consent. Participants' confidentiality was ensured by excluding personal identification (such as names) from forms used to collect study information and by storing study documents and samples in a secure location. Study staff members were trained on issues of confidentiality, good clinical practice, research ethics and protection of human subjects.

### Study procedures

HIV-1 seronegative, non-pregnant women aged 18–44 years working in bars, hotels, and other food and recreational facilities in the three towns of Geita, Shinyanga and Kahama in northwestern Tanzania were recruited into a 12 month microbicide feasibility study in 2008–2010. These towns are located near large-scale gold or diamond mines in which there are large populations of migratory male workers. The study population, inclusion criteria and procedures have been detailed previously [18]. Following the informed consent process, a face-to-face interview was conducted to elicit information on sociodemographic characteristics, sexual behaviour, contraceptive use, alcohol use and genital symptoms. Each participant was examined and samples for STIs and other reproductive tract infections (RTIs) were obtained, including a posterior fornix vaginal swab for *T. vaginalis* culture, a high lateral vaginal swab for assessing the vaginal microbiota (e.g. bacterial vaginosis), and a cervical swab for detection of *C. trachomatis* and *N. gonorrhoeae*. A blood sample was collected for testing for syphilis, HIV, and HSV-2, and a urine sample for pregnancy testing. Participants diagnosed with pelvic inflammatory disease (PID), vaginal discharge syndrome (VDS), or genital ulcer disease (GUD) were treated according to Tanzanian Ministry of Health syndromic STI management guidelines [14]. Participants were asked to return every three months for one year for a total of five visits. At follow-up visits, similar procedures were conducted with a face-to-face interview, gynaecological examination and blood draw for HIV and syphilis. However, testing for *C. trachomatis, N. gonorrhoeae*, *T. vaginalis* and HSV-2 was done only at enrolment and months six and 12.

If the participant had not previously been treated by syndromic management and had a positive laboratory test for *C. trachomatis, N. gonorrhoeae*, *T. vaginalis* or syphilis, they were traced and treated for the infection between study visits.

### Laboratory methods

Serum samples were tested for syphilis, HIV and HSV-2. Syphilis was diagnosed using a semi-quantitative RPR test (Immutrep Carbon Antigen, Omega Diagnostics, Alva, Scotland) and the *Treponema pallidum* particle agglutination assay (TPPA) (SERODIA, Fujirebio Inc., Tokyo, Japan). At enrolment, participants who were TPPA-positive and RPR-positive were considered to be prevalent cases of active syphilis, and participants who were TPPA-positive and had an RPR titre of at least 1∶8 were considered to have high-titre active syphilis. An incident case of syphilis was defined as follows: participants who were previously TPPA-negative and were TPPA-positive at a follow-up visit; or participants who were previously TPPA-positive and had a two-fold increase in RPR titre (to a titre of at least 1∶8) at a follow-up visit. HIV testing was performed in parallel using third generation Murex HIV 1.2.O (Abbott UK, Dartford, Kent, England) and Vironostika HIV Uniform II plus O (bioMerieux Bv, The Netherlands) enzyme-linked immunosorbent assays (ELISAs). Samples discrepant or indeterminate on ELISA were tested for P24 Antigen (Genetics Systems HIV-1 Ag EIA, Bio-rad Laboratories, Marnes-la-Coquette, France) and if positive were classified as HIV-positive. Samples negative for P24 antigen were tested by Western Blot (INNO-LIA, HIV I/II score, Innogenetics NV, Gent, Belgium). HSV-2 was detected using type-specific IgG ELISA (Kalon Biologicals Ltd., Guildford, UK.

A cotton-tipped swab was collected from the posterior fornix of the vagina for culture of *T. vaginalis* (InPouch, BioMed Diagnostics, San Jose, CA, USA), incubated at 36–37°C and read every 24 hours for 72 hours. Dacron swabs were collected from the endocervix and tested for *C. trachomatis* and *N. gonorrhoeae* by PCR (AMPLICOR, Roche Diagnostics, Branchburg, NJ, USA). All positive tests for *N. gonorrhoeae* were confirmed using specific primers to the 16S rDNA coding region using PCR in-house assays [19]. Vaginal microbiota were evaluated by microscopy of a Gram-stained vaginal smear using Nugent criteria which defines normal microbiota (scores 0–3), intermediate microbiota (scores 4–6) and bacterial vaginosis (scores 7–10) [20]. Candidiasis was diagnosed by wet mount microscopy.

### Statistical methods

Questionnaires were reviewed at site for completeness and consistency, and transported to a central data management centre. Double data entry was performed using DMSys software (SigmaSoft International, Chicago, IL, USA). Analysis was performed using Stata 11 (StataCorp, College Station, TX, USA).

Prevalence of infections and syndromes were calculated, and 95% confidence intervals were derived using Wilson's formula [21]. Odds ratio trends for *C. trachomatis, N. gonorrhoeae*, *T. vaginalis* and syndromes were estimated using logistic regression with random effects to account for repeated visits and within-women clustering. There were few incident syphilis cases (36 cases of incident syphilis; incidence rate was 4.8 (95% CI 3.5, 6.7) per 100 women-years), and we therefore restricted our analyses to prevalent high-titre active syphilis at enrolment only. Sensitivity, specificity and predictive values of PID and VDS (without curd-like discharge) for the detection of laboratory diagnosed STIs were calculated. We did not calculate the sensitivity, specificity and predictive values of GUD for the detection of syphilis as GUD can be caused by syphilis, but also by herpes, chancroid and lymphogranuloma venereum, and because serological syphilis may not always indicate a current active infection. Concurrent partnerships were defined as having more than one partner in the last three months.

We investigated associations with sociodemographic, behavioural and biological factors, over all visits using logistic regression with random effects for *C. trachomatis, N. gonorrhoeae* and *T. vaginalis*, and at enrolment using logistic regression for high-titre active syphilis. We adjusted *a priori* for visit month, town and age in all models. We built a multivariable model using a conceptual framework with three levels (sociodemographic, behavioural and biological). First, sociodemographic factors whose town- and age-adjusted associations were significant at p<0.10 were included in a multivariable model; stepwise backward selection (remove if p>0.05; re-enter if p<0.05), to remove those no longer independently associated, yielded our first core model. Second, behavioural factors were added to this core model one by one; those that were associated with the outcome at p<0.10, after adjusting for sociodemographic factors, were included in a multivariable model and stepwise backward selection (same criteria as above) was applied to the behavioural variables to yield our second core model. Third, biological factors were assessed in a similar way (third core model). We present results at each level of the framework, adjusted for more distal variables. Behavioural and biological factors which were measured at each visit were incorporated in a time-dependent manner, with the values at each visit included to assess the associations with the STIs at the same visit.

## Results

We enrolled 966 women (375 Geita, 306 Kahama, 285 Shinyanga) of median age 26 years (interquartile range: 22, 32 years). Only 121 (13%) women had progressed to secondary education, 428 (44%) worked as waitresses, 433 (46%) had been at their current facility for one year or less, 432 (45%) reported that they had ever engaged in transactional sex, 216 (24%) had concurrent partners, 84 (9%) were using oral contraceptives, and 136 (14%) were using the injectable contraceptive depot medroxyprogesterone acetate (DMPA). The proportions of women who attended visits at months 3, 6, 9 and 12 were 79%, 75%, 76% and 84%, respectively.

### Prevalence of laboratory diagnosed curable STIs

**Figure 1:**
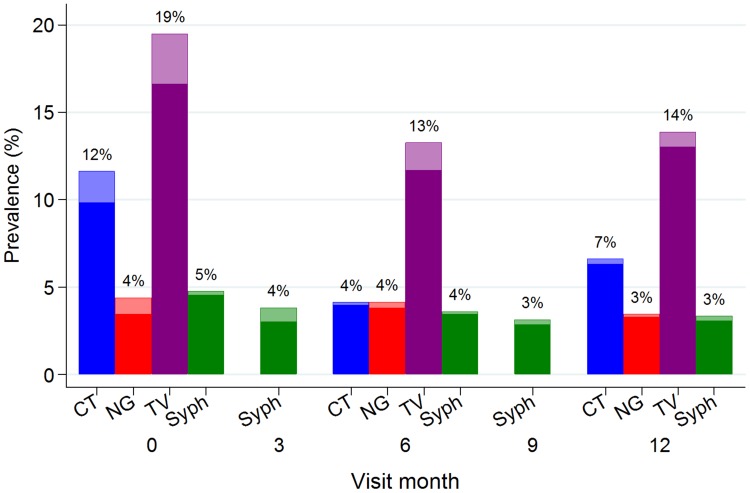
Prevalence of (a) curable sexually transmitted infections, and (b) syndromes diagnosed, by visit month, in a cohort of women at increased risk for HIV acquisition in northwestern Tanzania. Lighter shaded section of each bar represents those who were diagnosed by syndromic management. Values shown at the top of the bars are the overall prevalence (%). CT = *Chlamydia trachomatis*, NG = *Neisseria gonorrhoeae*, TV = *Trichomonas vaginalis*, syph = high-titre active syphilis.

At enrolment, the prevalences of *C. trachomatis*, *N. gonorrhoeae*, and *T. vaginalis* were 111/956 (12%), 42/955 (4%), and 184/945 (19%), respectively. The prevalence of active syphilis was 87/965 (9%) and the prevalence of high-titre active syphilis was 46/965 (5%). Overall, 313/939 (33%) women had at least one of these four STIs. The majority of these infections were not diagnosed with the corresponding syndrome and therefore most participants were not treated at enrolment. Figure 1 shows the curable STI prevalences by visit, illustrating the study design in which syphilis was tested for at every visit and *C. trachomatis*, *N. gonorrhoeae*, and *T. vaginalis* were tested for at enrolment, six and 12 months only. There were significant decreases over the follow-up period in the prevalence of *C. trachomatis* and *T. vaginalis* (odds ratio [OR] test for linear trend per month 0.94 [95% confidence interval [CI] 0.91, 0.97] and 0.95 [0.93, 0.98], respectively; both p<0.001) (Table 1).

**Table 1:** Trend in prevalence of laboratory-diagnosed curable sexually transmitted infections and syndromes diagnosed over the 12 month follow-up period in a cohort of women at increased risk for HIV acquisition in northwestern Tanzania.

	Prevalence at enrolment (%; 95% CI)	OR (95% CI) for linear trend (per month)	P-value
Infections			
*C. trachomatis*	111/956 (12%; 10, 14)	0.94 (0.91,0.97)	<0.001
*N. gonorrhoeae*	42/955 (4%; 3, 6)	0.98 (0.94,1.02)	0.34
*T. vaginalis*	184/945 (19%; 17, 22)	0.95 (0.93,0.98)	<0.001
Syndromes			
Pelvic inflammatory disease	249/966 (26%; 23, 29)	0.85 (0.83,0.88)	<0.001
Vaginal discharge syndrome (without curd-like discharge)	108/966 (11%; 9, 13)	0.91 (0.88,0.94)	<0.001
Vaginal discharge syndrome (with curd-like discharge)	92/966 (10%; 9,12)	0.95 (0.92,0.98)	<0.001

CI = confidence interval; OR = odds ratio.

### Prevalence and performance of STI Syndromes

**Figure 2:**
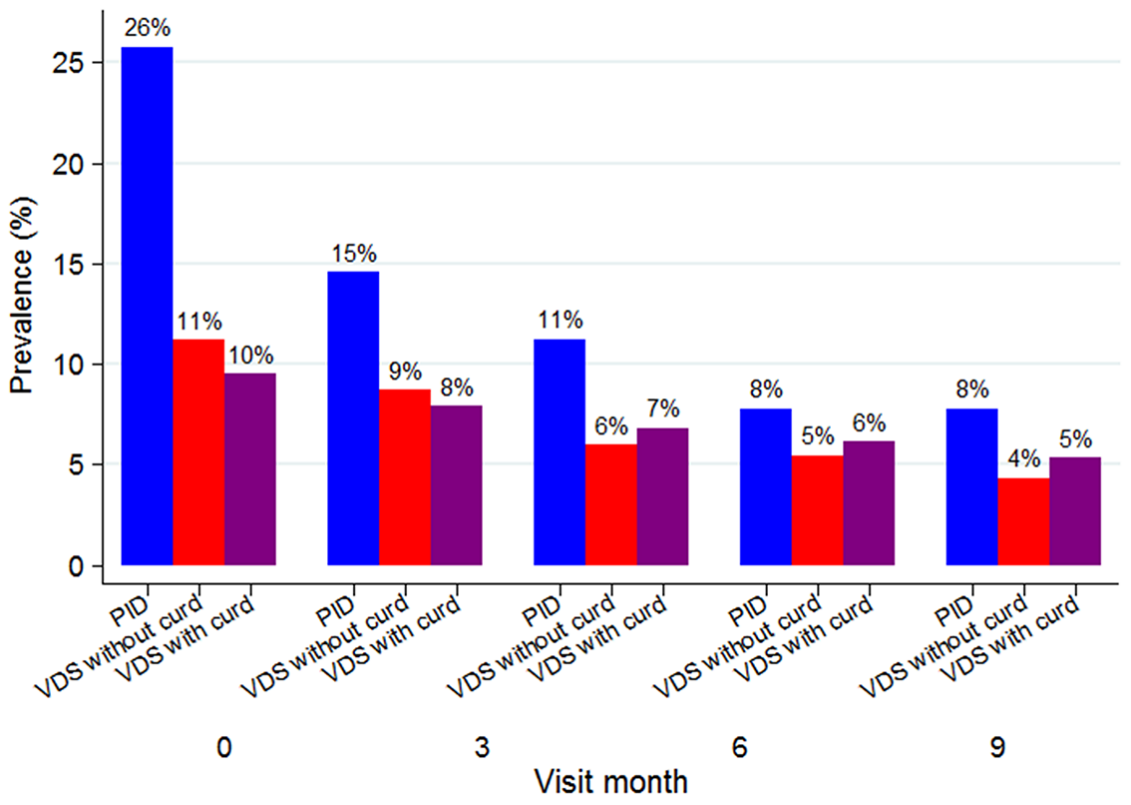
Prevalence of syndromes diagnosed, by visit month, in a cohort of women at increased risk for HIV acquisition in northwestern Tanzania. Values shown at the top of the bars are the prevalence (%). PID = pelvic inflammatory disease, VDS = vaginal discharge syndrome.

During the enrolment clinical interview, 539/962 (56%) participants reported genitourinary complaints, among whom itching was the most common complaint (64%), followed by abdominal pain during sexual intercourse (46%), vulvar burning (36%), dysuria (27%), abnormal vaginal discharge (25%) and genital ulcers or blisters (12%). On the enrolment genital examination, 500/956 (52%) participants had one or more clinical signs suggestive of an STI. The most common syndromic diagnosis was PID (26%), followed by VDS without curd-like discharge (11%), VDS with curd-like discharge (10%), and GUD (6%). At enrolment, 634 (66%) were negative for both PID and VDS (without curd-like discharge). Figure 2 shows the prevalence of syndromes by visit. There were significant decreases over the follow-up period for all syndromes (OR test for linear trend per month for PID = 0.85 [95% CI 0.83, 0.88], VDS without curd-like discharge 0.91 [0.88, 0.94], VDS with curd-like discharge 0.95 [0.92, 0.98] and GUD 0.87 [0.83, 0.91]; all p<0.001; Table 1).

Table 2 shows the sensitivity, specificity, and predictive values of syndromic management guidelines for PID and VDS (without curd-like discharge). The sensitivity and specificity of a PID diagnosis to detect *C. trachomatis*, *N. gonorrhoeae*, or bacterial vaginosis were 17% and 85%, respectively (similar results for each infection alone). The positive predictive value (PPV) of PID was low for *C. trachomatis* and *N. gonorrhoeae*, 12%, and 6% respectively, and higher for bacterial vaginosis, 49%. The negative predictive value (NPV) was high for *C. trachomatis* and *N. gonorrhoeae*, 93% and 96% respectively, and relatively lower for bacterial vaginosis, 55%. The sensitivity and specificity of VDS to detect *C. trachomatis*, *N. gonorrhoeae*, *T. vaginalis* or bacterial vaginosis were 9% and 94%, respectively (similar results for each infection alone). The PPV and NPV of VDS ranged from 7% and 96%, respectively, to 66% and 41%, respectively, for all four infections combined. The sensitivity, specificity, PPV and NPV of PID or VDS to detect either *C. trachomatis* or *N. gonorrhoeae* were 32%, 77%, 15%, and 90% respectively.

**Table 2:** Performance of syndromes for detection of laboratory-diagnosed sexually transmitted infections (STI) and bacterial vaginosis over the 12 month follow-up period in a cohort of women at increased risk for HIV acquisition in northwestern Tanzania.

	No. visits[1]	No. infected	No. with syndrome	No. cases detected by syndrome	Sensitivity	Specificity	Positive predictive value	Negative predictive value
**Pelvic inflammatory disease (PID) for**								
*C. trachomatis*	2279	183	389	45	25%	84%	12%	93%
*N. gonorrhoeae*	2277	92	388	25	27%	83%	6%	96%
**Bacterial vaginosis** (BV)	3968	1819	558	272	15%	87%	49%	55%
*C. trachomatis*, *N. gonorrhoeae* **or** BV	2373	1269	388	220	17%	85%	57%	47%
**Vaginal discharge syndrome without curd-like discharge (VDS) for**								
*C. trachomatis*	2279	183	182	20	11%	92%	11%	92%
*N. gonorrhoeae*	2277	92	182	12	13%	92%	7%	96%
*T. vaginalis*	2279	365	117	43	12%	93%	24%	85%
BV	3968	1819	289	150	8%	94%	52%	55%
*C. trachomatis, N. gonorrhoeae T. vaginalis* or BV[2]	2361	1417	182	121	9%	94%	66%	41%
**PID or VDS for**								
*C. trachomatis* **or** *N. gonorrhoeae*	2277	258	539	83	32%	77%	15%	90%

[1] The number of visits represent the number of visits with available test results and where clinical examination was performed. *C. trachomatis, N. gonorrhoeae*, and *T. vaginalis* were tested at baseline and 6 and 12 months, bacterial vaginosis was tested at every visit.

[2] Calculations were for visits when all infections were obtained by design (i.e. Enrolment, 6 and 12 months).

Low PPV meant that many participants were treated with antibiotics without an infection subsequently confirmed by laboratory diagnosis. Of the visits at which doxycycline (treatment for VDS and PID) or erythromycin (treatment for VDS and PID if pregnant) were prescribed, 809/904 (89%) did not have a laboratory diagnosis for *C. trachomatis*. Of the visits at which ciprofloxacin (treatment for VDS and PID) or ceftriaxone (treatment for VDS and PID if pregnant) were prescribed, 773/830 (93%) did not have a laboratory diagnosis for *N. gonorrhoeae*. Of the visits where metronidazole (treatment for VDS and PID) was prescribed, 632/782 (81%) did not have a laboratory diagnosis of *T. vaginalis* or bacterial vaginosis.

### Risk factors for laboratory-diagnosed STIs

Results from multivariate models for each level of the conceptual framework are shown in Tables S1–S4 in File S1, with results from the final model at each level reported in Table 3. Participants who had been working in their facility for a longer time were less likely to be infected with *C. trachomatis* (p-trend = 0.01; Table 3). Age was strongly associated with *C. trachomatis* infection, with younger participants being at higher risk (p-trend = 0.003). Participants who had more reported lifetime sex partners were more likely to be infected with C. *trachomatis* infection (p-trend = 0.005). There was evidence of an association between *C. trachomatis* infection and contraception use (p = 0.02); those using DMPA (adjusted[a]OR 2.21; CI 1.39–3.52) and condoms only (aOR 1.50; CI 1.01–2.24) were at higher risk compared to those who reported no contraception use. Participants who had at least one pregnancy in their lifetime had twice the odds of *C. trachomatis* infection (aOR 1.96; 95% CI 1.10, 3.50) compared with those who had never been pregnant. *C. trachomatis* infection was also associated with *N. gonorrhoeae* infection (aOR 2.21; CI 1.23–3.97).

**Table 3:** Associations of *C. trachomatis*, *N. gonorrhoeae*, *T. vaginalis* and active syphilis (high-titre) with sociodemographic, behavioural and biological factors in a cohort of women at increased risk for HIV acquisition in northwestern Tanzania.

	*C. trachomatis*			*N. gonorrhoeae*			*T. vaginalis*			Active syphilis (high-titre)		
	**No. positive/No. obs (%)**	**Visit month-, town- and age-adjusted OR**	**Adjusted OR (95% CI) [1]**	**No. positive/No. observations (%)**	**Visit month-, town- and age-adjusted OR**	**Adjusted OR (95% CI) [2]**	**No. positive/No. observations (%)**	**Visit month-, town- and age-adjusted OR**	**Adjusted OR (95% CI) [3]**	**No. positive/No. observations (%)**	**Town- and age-adjusted OR**	**Adjusted OR (95% CI) [4]**
**SOCIODEMOGRAPHIC FACTORS**												
**Town**			**P = 0.37**			**P = 0.55**			**P = 0.75**			**P = 0.19**
Geita	62/863 (7%)	1	**1**	32/862 (4%)	1	**1**	137/862 (16%)	**1**	**1**	25/375 (7%)	1	**1**
Kahama	69/723 (10%)	1.33	**1.30 (0.89, 1.90)**	36/722 (5%)	1.34	**1.34 (0.78, 2.29)**	123/707 (17%)	**1.06**	**1.15 (0.76, 1.75)**	13/306 (4%)	0.63	**0.76 (0.38,1.54)**
Shinyanga	52/693 (8%)	1.23	**1.21 (0.81, 1.82)**	24/693 (3%)	1.07	**1.07 (0.59, 1.94)**	105/710 (15%)	**0.88**	**1.00 (0.65, 1.54)**	8/284 (3%)	0.40	**0.48 (0.21,1.10)**
**Job**			P = 0.15			P = 0.42			P = 0.38			P = 0.38
Waitress	100/940 (11%)	1	1	49/939 (5%)	1	1	176/943 (19%)	1	1	25/428 (6%)	1	1
Independent food vendor	30/519 (6%)	0.63	0.69 (0.44, 1.07)	17/519 (3%)	0.72	0.72 (0.39, 1.34)	69/513 (13%)	0.62	0.73 (0.45, 1.18)	8/200 (4%)	0.63	0.65 (0.28,1.50)
Other	53/820 (6%)	0.72	0.76 (0.52, 1.10)	26/819 (3%)	0.74	0.74 (0.43, 1.26)	120/823 (15%)	0.72	0.82 (0.54, 1.23)	13/337 (4%)	0.60	0.64 (0.31,1.31)
**Duration working in facility type, years**			**P = 0.04**			P = 0.90			P = 0.24			P = 0.18
			**p-trend = 0.01**			p-trend = 0.67			p-trend = 0.11			p-trend = 0.44
(0,1]	101/987 (10%)	1	**1**	47/985 (5%)	1	1	173/979 (18%)	1	1	21/433 (5%)	1	1
(1,5]	52/750 (7%)	0.71	**0.71 (0.49, 1.02)**	29/750 (4%)	0.90	0.90 (0.53, 1.50)	119/745 (16%)	0.85	0.89 (0.59, 1.33)	19/317 (6%)	1.25	1.29 (0.66,2.50)
>5	21/479 (4%)	0.57	**0.57 (0.34, 0.97)**	13/479 (3%)	0.89	0.89 (0.43, 1.82)	60/489 (12%)	0.58	0.64 (0.38, 1.08)	5/187 (3%)	0.48	0.50 (0.17,1.48)
**Age, years**			**P = 0.001**			**P<0.001**			**P = 0.82**			**P = 0.16**
			**p-trend = 0.003**			**p-trend<0.001**			**p-trend = 0.66**			**p-trend = 0.91**
<20	20/176 (11%)	1	**1**	13/176 (7%)	1	**1**	27/175 (15%)	1	**1**	5/88 (6%)	1	**1**
20–24	70/666 (11%)	0.92	**0.97 (0.56, 1.71)**	33/665 (5%)	0.62	**0.62 (0.29, 1.31)**	118/656 (18%)	1.20	**1.27 (0.63, 2.55)**	18/304 (6%)	1.12	**1.18 (0.42,3.32)**
25–29	58/579 (10%)	0.89	**1.05 (0.59, 1.87)**	31/578 (5%)	0.68	**0.68 (0.32, 1.45)**	96/579 (17%)	1.09	**1.15 (0.55, 2.43)**	6/246 (2%)	0.45	**0.46 (0.13,1.57)**
≥30	35/858 (4%)	0.35	**0.45 (0.24, 0.84)**	15/858 (2%)	0.21	**0.21 (0.09, 0.49)**	124/869 (14%)	0.90	**1.04 (0.49, 2.22)**	17/327 (5%)	1.06	**1.16 (0.41,3.32)**
**Education level achieved**			P = 0.08			P = 0.26			**P = 0.001**			**P<0.001**
			p-trend = 0.75			p-trend = 0.85			**p-trend<0.001**			
No education or incomplete primary	66/678 (10%)	1	1	23/677 (3%)	1	1	146/675 (22%)	1	**1**	27/298 (9%)	1	**1**
Complete primary	86/1312 (7%)	0.67	0.71 (0.50, 1.02)	59/1311 (5%)	1.43	1.43 (0.83, 2.45)	182/1312 (14%)	0.51	**0.52 (0.36, 0.77)**	19/666 (3%)	0.31	**0.31 (0.17,0.57)**
≥Secondary	31/287 (11%)	1.01	1.09 (0.67, 1.76)	10/287 (3%)	0.89	0.89 (0.39, 2.02)	36/290 (12%)	0.40	**0.40 (0.22, 0.74)**	[5]		
**Marital status**			P = 0.42			P = 0.06			**P = 0.001**			P = 0.98
Married	27/511 (5%)	1	1	12/510 (2%)	1	1	51/515 (10%)	1	**1**	9/203 (4%)	1	1
Separated/divorced/widowed	88/1156 (8%)	1.30	1.24 (0.78, 1.96)	54/1155 (5%)	1.76	1.76 (0.88, 3.53)	216/1156 (19%)	2.47	**2.42 (1.49, 3.93)**	24/482 (5%)	1.12	1.09 (0.49,2.44)
Single	68/612 (11%)	1.51	1.41 (0.84, 2.39)	26/612 (4%)	0.96	0.96 (0.43, 2.15)	98/608 (16%)	1.71	**1.96 (1.08, 3.57**)	13/280 (5%)	0.87	1.10 (0.40,2.98)
**BEHAVIOURAL FACTORS AT ENROLMENT**												
**AUDIT score [6]**			P = 0.38			**P = 0.04**			P = 0.79			P = 0.59
Non-drinker or low risk drinking	149/1945 (8%)	1	1	71/1944 (4%)	1	**1**	307/1946 (16%)	1	1	37/816 (5%)	1	1
Harmful or hazardous drinking	33/323 (10%)	1.26	1.22 (0.79, 1.86)	21/322 (7%)	1.83	**1.83 (1.03, 3.25)**	54/323 (17%)	1.03	0.94 (0.57, 1.53)	8/144 (6%)	1.27	0.76 (0.28,2.11)
**Age at first sex, years**			P = 0.17			P = 0.61			P = 0.06			P = 0.36
<16	61/663 (9%)	1	1	31/663 (5%)	1	1	129/664 (19%)	1	1	21/286 (7%)	1	1
≥16	96/1432 (7%)	0.74	0.77 (0.54, 1.11)	53/1430 (4%)	0.85	0.87 (0.52, 1.46)	205/1436 (14%)	0.61	0.69 (0.47, 1.02)	21/600 (4%)	0.47	0.71 (0.34,1.48)
**N lifetime sex partners**			**P = 0.03**			P = 0.89			P = 0.95			P = 0.59
			**p-trend = 0.005**			p-trend = 0.61			p-trend = 0.60			p-trend = 0.89
0–4	59/930 (6%)	1	**1**	36/930 (4%)	1	1	138/933 (15%)	1	1	14/390 (4%)	1	1
5–9	41/483 (8%)	1.41	**1.52 (0.99, 2.36)**	22/483 (5%)	1.21	1.13 (0.61, 2.08)	81/486 (17%)	1.19	1.01 (0.63, 1.62)	9/210 (4%)	1.10	0.72 (0.28,1.87)
≥10	36/405 (9%)	1.49	**1.48 (0.93, 2.36)**	18/404 (4%)	1.13	0.93 (0.47, 1.85)	65/404 (16%)	1.17	0.92 (0.55, 1.53)	10/168 (6%)	1.59	0.47 (0.15,1.50)
Do not remember	46/451 (10%)	1.76	**1.91 (1.23, 2.96)**	16/450 (4%)	0.94	0.84 (0.43, 1.67)	80/446 (18%)	1.33	1.09 (0.67, 1.78)	13/193 (7%)	2.01	0.99 (0.39,2.49)
**TIME-VARYING BEHAVIOURAL FACTORS**												
**N sex partners in last 3 mths**			P = 0.59			P = 0.41			P = 0.78			P = 0.28
			p-trend = 0.30			p-trend = 0.29			p-trend = 0.59			p-trend = 0.13
0	126/1803 (7%)	1	1	64/1802 (4%)	1	1	274/1804 (15%)	1	1	22/693 (3%)	1	1
2	34/316 (11%)	1.41	1.21 (0.77, 1.89)	20/315 (6%)	1.66	1.50 (0.84, 2.69)	56/315 (18%)	1.04	0.99 (0.65, 1.52)	16/164 (10%)	3.34	0.14 (0.01,1.97)
≥3	21/145 (14%)	1.81	1.27 (0.71, 2.27)	8/145 (6%)	1.41	1.18 (0.50, 2.78)	33/146 (23%)	1.41	1.22 (0.69, 2.15)	7/93 (8%)	2.15	0.08 (0.00,1.41)
**Concurrent sex partners in last 3 mths**			P = 0.30			P = 0.33			P = 0.98			**P = 0.004**
No	126/1810 (7%)	1	1	65/1809 (4%)	1	1	276/1811 (15%)	1	1	21/687 (3%)	1	**1**
Yes	49/402 (12%)	1.58	1.24 (0.83, 1.87)	23/401 (6%)	1.44	1.33 (0.76, 2.35)	75/401 (19%)	1.07	0.99 (0.67, 1.48)	18/216 (8%)	2.90	**2.71 (1.39,5.28)**
**Transactional sex in last 3 months**			P = 0.90			P = 0.11			P = 0.20			P = 0.97
No	122/1753 (7%)	1	1	60/1753 (3%)	1	1	257/1760 (15%)	1	1	24/599 (4%)	1	1
Yes	61/522 (12%)	1.29	1.03 (0.69, 1.52)	32/520 (6%)	1.69	1.55 (0.91, 2.63)	108/515 (21%)	1.38	1.26 (0.88, 1.81)	22/362 (6%)	1.45	1.01 (0.48,2.12)
**Contraception**			**P = 0.02**			P = 0.37			P = 0.53			P = 0.89
None of these	54/964 (6%)	1	**1**	31/964 (3%)	1	1	155/974 (16%)	1	1	19/376 (5%)	1	1
Condom only	68/725 (9%)	1.41	**1.50 (1.01, 2.24)**	34/725 (5%)	1.17	1.18 (0.69, 2.02)	127/719 (18%)	1.14	1.02 (0.72, 1.46)	17/338 (5%)	0.89	0.76 (0.35,1.65)
Pill (+/−condoms)	16/196 (8%)	1.42	**1.51 (0.82, 2.79)**	8/196 (4%)	1.24	1.27 (0.54, 3.01)	31/193 (16%)	1.00	0.99 (0.54, 1.80)	4/84 (5%)	0.94	0.90 (0.25,3.23)
Injectable (DMPA; +/−condoms)	38/320 (12%)	2.04	**2.21 (1.39, 3.52)**	18/318 (6%)	1.69	1.66 (0.86, 3.21)	39/320 (12%)	0.75	0.69 (0.41, 1.14)	4/135 (3%)	0.62	0.57 (0.18,1.79)
Other hormonal contraceptives	7/73 (10%)	1.67	**1.76 (0.75, 4.13)**	1/73 (1%)	0.35	0.34 (0.04, 2.75)	13/72 (18%)	1.35	1.37 (0.55, 3.42)	2/31 (6%)	1.29	0.90 (0.11,7.36)
**BIOLOGICAL FACTORS AT ENROLMENT**												
**Ever pregnant**			**P = 0.02**			**P = 0.003**			P = 0.69			P = 0.59
No	22/318 (7%)	1	**1**	8/318 (3%)	1	**1**	55/314 (18%)	1	1	8/151 (5%)	1	1
Yes	161/1959 (8%)	1.96	**1.96 (1.10, 3.50)**	84/1957 (4%)	3.73	**3.39 (1.41, 8.16)**	310/1963 (16%)	0.93	0.89 (0.51, 1.57)	38/813 (5%)	1.03	0.76 (0.28,2.03)
**TIME-VARYING BIOLOGICAL FACTORS**												
**Current HSV-2 infection**			P = 0.42			P = 0.27			P = 0.46			P = 0.69
Negative	50/503 (10%)	1	1	17/502 (3%)	1	1	66/497 (13%)	1	1	11/249 (4%)	1	1
Positive (from baseline)	128/1720 (7%)	0.95	0.80 (0.54, 1.18)	71/1719 (4%)	1.72	1.43 (0.78, 2.63)	292/1726 (17%)	1.64	1.29 (0.82, 2.03)	35/715 (5%)	1.19	0.84 (0.37,1.95)
Positive (seroconverted during follow-up)	5/53 (9%)	1.41	1.26 (0.45, 3.53)	4/53 (8%)	2.65	2.59 (0.73, 9.19)	7/53 (13%)	1.00	0.84 (0.28, 2.53)	[7]		
**Current syphilis status ** [8]			P = 0.61			P = 0.47			P = 0.08	-	-	-
Never infected	153/1861 (8%)	1	1	81/1859 (4%)	1	1	278/1857 (15%)	1	1			
Previous infection	10/227 (4%)	0.70	0.73 (0.37, 1.45)	4/227 (2%)	0.53	0.54 (0.18, 1.59)	35/230 (15%)	1.20	1.01 (0.58, 1.77)			
Active infection	20/190 (11%)	1.35	1.09 (0.62, 1.91)	7/190 (4%)	0.88	0.85 (0.36, 2.01)	52/191 (27%)	2.54	1.85 (1.08, 3.17)			
**Current candida status**			P = 0.20			P = 0.70			P = 0.73			P = 0.29
Negative	178/2152 (8%)	1	1	85/2150 (4%)	1	1	346/2153 (16%)	1	1	43/921 (5%)	1	1
Positive	5/118 (4%)	0.56	0.57 (0.22, 1.45)	5/118 (4%)	1.05	1.22 (0.45, 3.30)	17/118 (14%)	0.99	1.13 (0.57, 2.22)	3/35 (9%)	1.40	2.13 (0.58,7.83)
**Current ** ***T. vaginalis*** ** infection**			P = 0.87			P = 0.34	-	-	-			**P = 0.001**
Negative	145/1896 (8%)	1	1	72/1894 (4%)	1	1				26/760 (3%)	1	**1**
Positive	34/362 (9%)	1.12	0.97 (0.63, 1.48)	18/362 (5%)	1.30	1.33 (0.75, 2.36)				20/184 (11%)	3.43	**3.27 (1.66,6.43)**
**Current ** ***C. trachomatis*** ** infection**		-	-			**P = 0.02**			P = 0.86			P = 0.73
Negative				75/2094 (4%)	1	**1**	329/2080 (16%)	1	1	41/844 (5%)	1	1
Positive				17/183 (9%)	2.38	**2.19 (1.19, 4.05)**	34/180 (19%)	1.21	1.05 (0.63, 1.75)	5/111 (5%)	1.08	0.83 (0.27,2.50)
**Current ** ***N. gonorrhoeae*** ** infection**			**P = 0.01**	-	-	-			P = 0.62			P = 0.21
Negative	166/2186 (8%)	1	**1**				345/2169 (16%)	1	1	43/912 (5%)	1	1
Positive	17/92 (18%)	2.29	**2.21 (1.23, 3.97)**				18/90 (20%)	1.37	1.20 (0.59, 2.44)	3/42 (7%)	1.83	2.51 (0.68,9.28)
**Current vaginal microbiota**			P = 0.07			P = 0.38			**P<0.001**			P = 0.08
Negative	50/830 (6%)	1	1	24/828 (3%)	1	1	72/829 (9%)	1	**1**	10/357 (3%)	1	1
Indeterminate	42/397 (11%)	1.79	1.70 (1.07, 2.69)	18/397 (5%)	1.49	1.38 (0.71, 2.68)	103/396 (26%)	4.70	**4.37 (2.84, 6.72)**	7/161 (4%)	1.58	0.62 (0.19,1.97)
Bacterial vaginosis	91/1051 (9%)	1.36	1.34 (0.92, 1.97)	50/1051 (5%)	1.53	1.44 (0.84, 2.45)	190/1053 (18%)	2.37	**2.28 (1.58, 3.30)**	29/446 (7%)	2.47	1.72 (0.78,3.80)
**Currently HIV-positive**			P = 0.51		(not estimable)	-			P = 0.82	[9]		
Negative	180/2241 (8%)	1	1	92/2239 (4%)			360/2242 (16%)	1	1			
Positive	3/38 (8%)	1.39	1.55 (0.45, 5.34)	0/38 (0%)			5/37 (14%)	0.99	0.86 (0.25, 3.00)			
**Currently have diagnosis of GUD**			P = 0.15			P = 0.22			P = 0.73			P = 0.92
Negative	168/2200 (8%)	1	1	85/2198 (4%)	1	1	352/2193 (16%)	1	1	44/900 (5%)	1	1
Positive	15/78 (19%)	1.99	1.71 (0.86, 3.41)	7/78 (9%)	1.77	1.83 (0.73, 4.62)	12/71 (17%)	0.89	0.86 (0.37, 1.99)	2/55 (4%)	0.80	1.08 (0.22,5.20)
**Currently have a diagnosis of VDS**			P = 0.59			P = 0.31			P = 0.13			P = 0.76
No	163/2097 (8%)	1	1	80/2095 (4%)	1	1	322/2102 (15%)	1	1	42/857 (5%)	1	1
Yes	20/182 (11%)	1.06	0.85 (0.48, 1.52)	12/182 (7%)	1.50	1.47 (0.71, 3.03)	43/177 (24%)	1.68	1.49 (0.90, 2.47)	4/108 (4%)	0.78	0.83 (0.26,2.70)

Note: *C. trachomatis*, *N. gonorrhoeae* and *T. vaginalis* data from all visits were used in the analysis. For active syphilis (high-titre), data only from the enrolment visit were used since there were very few incident infections. “1” denotes the reference category throughout.

[1] ORs for sociodemographic variables adjusted for visit month, town, age and duration working in facility type; ORs for behavioural variables adjusted for these sociodemographic variables, number of lifetime partners and contraception; ORs for biological variables adjusted for these sociodemographic and behavioural variables, ever pregnant and gonorrhoea status (the results for these variables are shown in bold). Full results from each level shown in Table S1 in File S1.

[2] ORs for sociodemographic variables adjusted for visit month, town and age; ORs for behavioural variables adjusted for these sociodemographic variables and AUDIT; ORs for biological variables adjusted for these sociodemographic and behavioural variables, ever pregnant and chlamydia status (the results for these variables are shown in bold). Full results from each level shown in Table S2 in File S1.

[3] ORs for sociodemographic variables adjusted for visit month, town, age, education and marital status; ORs for behavioural variables adjusted for these sociodemographic variables (no behavioural variables included); ORs for biological variables adjusted for these sociodemographic and behavioural variables and current vaginal microbiota assessed by Nugent score (the results for these variables are shown in bold). Full results from each level shown in Table S3 in File S1.

[4] ORs for sociodemographic variables adjusted for visit month, town, age and education; ORs for behavioural variables adjusted for these sociodemographic variables and whether had concurrent partners in the last 3 months; ORs for biological variables adjusted for these sociodemographic and behavioural variables and *T. vaginalis* status (the results for these variables are shown in bold). Full results from each level shown in Table S4 in File S1.

[5] Combined complete primary and secondary since only two women who attended secondary school had active syphilis (high-titre) at enrolment.

[6] Based on responses to ten AUDIT questions. Scores based on responses to each question: 0–7 = non-drinker or low-risk; ≥8 harmful or hazardous drinking.

[7] Combined positive (from baseline) and positive (seroconverted during follow-up).

[8] Current syphilis status is defined as follows: RPR negative/RPR positive and TPPA negative = never infected (includes biological false positives); RPR negative and TPPA positive = previous infection; RPR positive and TPPA positive = active infection.

[9] All women HIV-negative at enrolment as per eligibility criteria.

Younger women were also at higher risk of *N. gonorrhoeae* infection (p-trend<0.001; Table 3). There was some evidence that harmful or hazardous drinking, compared to no or low-risk drinking, was associated with *N. gonorrhoeae* infection (aOR 1.83; CI 1.03, 3.25). Participants who had at least one pregnancy in their lifetime had more than three times the odds of *N. gonorrhoeae* infection (aOR 3.39; CI 1.41, 8.16). *N. gonorrhoeae* was strongly associated with *C. trachomatis* infection (aOR 2.19; CI 1.19, 4.05).

Higher education level was associated with lower odds of *T. vaginalis* infection (p-trend<0.001; Table 3). Higher *T. vaginalis* prevalence was found in divorced, separated or widowed (aOR 2.42; 1.49, 3.93) and single (aOR1.96; 1.08, 3.57) women compared to married women. Current intermediate vaginal microbiota (aOR 4.37; 2.84, 6.72) and bacterial vaginosis (aOR 2.28; 1.58, 3.30) were strongly associated with *T. vaginalis* infection. There was weak evidence of an association between *T. vaginalis* and syphilis (p = 0.08), in particular for active syphilis (aOR 1.85; 1.08, 3.17).

Higher education levels were also associated with lower prevalence of high-titre active syphilis infection at enrolment (aOR 0.31; 0.17, 0.57 for those who completed primary school compared with those who did not; Table 3). Participants who reported concurrent sex partners in the past three months had higher odds of high-titre active syphilis (aOR 2.71; 1.39, 5.28). *T. vaginalis* infection (aOR 3.27; 1.66, 6.43) was strongly associated with high-titre active syphilis infection.

## Discussion

The prevalences of curable STIs were high indicating that these infections are a major public health problem among women working in bars, hotels, and other food and recreational facilities near large-scale gold and diamond mines in northwestern Tanzania. Our findings were consistent with previous studies from similar populations in Tanzania, and indicate continued high burden of these infections [9], [10], [12]. *T. vaginalis* was the most prevalent infection at enrolment and throughout the study period. Further, while culture is considered the gold standard for detection of *T. vaginalis*, the sensitivity of culture has been reported to be 75% to 89% compared to nucleic acid amplification tests [45], [46], and therefore the prevalence of *T. vaginalis* in this study population may be underestimated. *T. vaginalis* infection has been associated with pelvic inflammatory disease, endometritis, adverse pregnancy outcomes, and HIV acquisition [22]–[25]. There is also evidence that HIV infection is associated with *T. vaginalis* acquisition [26] Therefore, *T. vaginalis* is an important infection to target in prevention efforts as this synergy may contribute in continued expansion of the HIV epidemic in this population [26].

We observed a significant decrease in the prevalence of *C. trachomatis* and *T. vaginalis* over the one-year follow-up period, consistent with the provision of behavioural counselling, condom provision and free STI treatment as seen in other studies [12]. However, we did not observe a significant decrease in syphilis which may reflect a slow decline in RPR titre after treatment, or serofast reactions in which nontreponemal antibodies persist for a long period of time [27].

Due to a low sensitivity of syndromic management to detect laboratory-diagnosed infections in this population at increased risk for HIV infection, most infections were missed by this approach. Thus, the majority of these infections would have gone untreated in a syndromic management setting where laboratory testing was not routinely done. Poor sensitivity of syndromic management has been documented in other settings, for example among female sex workers in India [28]. It is important to note that syndromic STI management guidelines were not developed as a screening tool for asymptomatic patients. Nevertheless, in many resource-limited settings, this is the only STI management tool available – even to populations at increased risk for STIs and HIV. Furthermore, many research cohorts rely on syndromic management to treat STIs in their study populations until laboratory results are available. The results of this study are a reminder that this approach will miss a high proportion of cases, and in the interim period between study visit and laboratory diagnosis, transmission and sequelae of STIs may occur. Interestingly, a recent study among sex workers in South Africa has shown that cervicovaginal inflammatory cytokines did not differ between women with and without symptoms; and in both groups inflammatory cytokines were elevated [17]. Previous studies have suggested that elevated inflammatory cytokines may facilitate HIV transmission [29], and, thus, women with asymptomatic STIs may be as susceptible to HIV infection as those with symptoms.

This study also revealed the poor PPV of syndromic management, and as a consequence, a high proportion of participants in our study were given antibiotics that they did not need. In addition to the costs associated with over-treatment of STIs in a resource-limited setting, over-use of antibiotics is a major concern as resistance to *N. gonorrhoeae* has become widespread [30], [31] and there is evidence of fluoroquinolone resistance in East Africa [32], [33]. Furthermore, the first line treatment for *N. gonorrhoeae* in the Tanzanian Ministry of Health guidelines is ciprofloxacin. Research is urgently needed to evaluate fluoroquinolone resistance to *N. gonorrhoeae* in northwestern Tanzania. Although we found a relatively low prevalence of *N. gonorrhoeae* in our study, we found no evidence of a decrease in *N. gonorrhoeae* over the follow-up period, and this may have been due to treatment resistance. An important caveat is that while *C. trachomatis, N. gonorrhoeae* and bacterial vaginosis are found in some cases of PID, the infectious cause of PID is often unknown[34]; therefore, the absence of these pathogens does not necessarily signify the absence of PID, and does not indicate that treatment was not needed. Overall, our findings show that syndromic management is not effective for controlling STIs in this population at increased risk of HIV infection, and there is an urgent need to develop focused interventions and programmes to prevent, identify and treat STIs effectively in this population.

Our risk factor analysis identified women at greatest risk for STIs in this population and who may benefit from focused interventions. Young women were at increased risk for *C. trachomatis* and *N. gonorrhoeae* infection, and these infections were associated with each other. Lower levels of education were a risk factor for both *T. vaginalis* and high-titre active syphilis, and these infections were associated with each other. Married women were at lower risk of *T. vaginalis*. Sexual and other behavioural indices, such as higher total number of lifetime partners, concurrent sex partners and harmful alcohol use were associated with curable STIs. Overall, risk factors for curable STIs identified in this cohort were as expected and consistent with the literature [35]–[38]. One exception was the finding that women who had at least one pregnancy in their lifetime were at higher risk for *C. trachomatis* and *N. gonorrhoeae* infection; it is possible that a past pregnancy may be a proxy for past unprotected sex. Additionally, women who had recently started work in a recreational or food facility were at higher risk for *C. trachomatis* infection than those who had been there for a year or more. There is evidence suggesting partial immunity against reinfection with *C. trachomatis* [39]; therefore women entering into a high risk occupation may be at higher risk of infection than those who have been working in a high-risk setting for several years. It is also possible that some behaviour change may account for this difference, with women who have been working in a facility for a longer time possibly having fewer sexual partners or using condoms more often; however we found no evidence for this in this cohort.

Women in this study using DMPA were at higher risk for *C. trachomatis* infection. Several longitudinal studies have found an association between DMPA use and cervical infection with either *C. trachomatis* or *N. gonorrhoeae* [40]–[42], as well as HIV acquisition [43], although these findings have not been consistent [44]. While *C. trachomatis* may lie on the causal pathway between DMPA and HIV acquisition, DMPA may also increase susceptibility to both infections. It is also possible that women who use DMPA may have different sexual behaviour (e.g. use less condoms) than those who do not use DMPA, which may increase risk for STIs. Indeed, women using hormonal contraceptives have been shown to use condoms less frequently than women not using hormonal contraception [42].

The foundation of STI prevention and control is early diagnosis and treatment, including the treatment of partners. Yet, sensitive screening tests for STIs are expensive and rarely available in resource-limited settings. Affordable, rapid point-of-care tests would be valuable tools for detection and treatment of these infections in this population, and should be a priority for development [47]. A modelling study using data from a sex worker project in Benin suggested that rapid point-of-care screening tests for cervical infection due to *C. trachomatis* and *N. gonorrhoeae* may be more cost effective than a syndromic approach because of higher sensitivity and specificity [48]. Of the potential interventions available, there is good evidence that risk reduction counselling and condom promotion reduce STI risk [49]. Structural interventions, such as economic interventions or tackling gender disadvantage, are promising interventions to address main underlying factors in this population, but are not well evaluated [49].

In summary, we found a high prevalence of STIs among women who work in bars, hotels, and other food and recreational facilities near large-scale gold and diamond mines in northwestern Tanzania. This population is at increased risk for HIV infection in addition to reproductive health morbidities caused by STIs. Syndromic diagnosis, dependent on signs and symptoms of STIs, was poorly predictive of laboratory-diagnosed STIs. Untreated STIs in this population will translate into a high risk of STIs for male partners, sexual partners of those men, and infants who may become congenitally infected. The importance of the treatment errors and missed opportunities for cure should not be under-estimated. Innovative strategies to reduce the burden of STIs are urgently needed, and affordable rapid-point-of-care tests should be a priority for development.

## Supporting Information

File S1 **Supporting tables.** **Table S1**, Final sociodemographic-, behavioural- and biological-level model results for *Chlamydia trachomatis* (adjusted odds ratio and 95% CI). **Table S2**, Final sociodemographic-, behavioural- and biological-level model results for *Neisseria gonorrhoeae* (adjusted odds ratio and 95% CI). **Table S3**, Final sociodemographic-, behavioural- and biological-level model results for *Trichomonas vaginalis* (adjusted odds ratio and 95% CI). **Table S4**, Final sociodemographic-, behavioural- and biological-level model results for active syphilis (high titre) (adjusted odds ratio and 95% CI).
(DOCX)
