# Supplementary material for: Epidemiology of Curable Sexually Transmitted Infections among Women at Increased Risk for HIV in Northwestern Tanzania: Inadequacy of Syndromic Management
Source: PLoS One. 2014 Jul 15;9(7):e101221. doi: 10.1371/journal.pone.0101221 (PMC4099080; doi:10.1371/journal.pone.0101221)
Supplement: File S1 — Supporting tables. Table S1, Final sociodemographic-, behavioural- and biological-level model results for Chlamydia trachomatis (adjusted odds ratio and 95% CI). Table S2, Final sociodemographic-, behavioural- and biological-level model results for Neisseria gonorrhoeae (adjusted odds ratio and 95% CI). Table S3, Final sociodemographic-, behavioural- and biological-level model results for Trichomonas vaginalis (adjusted odds ratio and 95% CI). Table S4, Final sociodemographic-, behavioural- and biological-level model results for active syphilis (high titre) (adjusted odds ratio and 95% CI). (DOCX) [file pone.0101221.s001.docx]

**Table S1. Final sociodemographic-, behavioural- and biological-level model results for *Chlamydia trachomatis* (adjusted odds ratio and 95% CI).**

|  | **Final sociodemographic-level model [1]** | **Final behavioural-level model [2]** | **Final biological-level model [3]** |
| --- | --- | --- | --- |
| **SOCIO-DEMOGRAPHIC CHARACTERISTICS AT ENROLMENT** | |  |  |
| **Town** | **P= 0.37** | **P= 0.64** | **P= 0.68** |
| Geita | **1 (reference)** | **1 (reference)** | **1 (reference)** |
| Kahama | **1.30 (0.89, 1.90)** | **1.12 (0.76, 1.66)** | **1.10 (0.74, 1.64)** |
| Shinyanga | **1.21 (0.81, 1.82)** | **1.22 (0.80, 1.85)** | **1.21 (0.79, 1.83)** |
| **Job** | P= 0.15 | P= 0.67 | P= 0.70 |
| Waitress | 1 (reference) | 1 (reference) | 1 (reference) |
| Mamalishe | 0.69 (0.44, 1.07) | 0.84 (0.53, 1.34) | 0.84 (0.53, 1.35) |
| Other | 0.76 (0.52, 1.10) | 0.86 (0.59, 1.26) | 0.87 (0.60, 1.28) |
| **Duration working in facility type, years** | **P= 0.04** | **P= 0.02** | **P= 0.02** |
| ≤1 | **1 (reference)** | **1 (reference)** | **1 (reference)** |
| >1-≤5 | **0.71 (0.49, 1.02)** | **0.69 (0.48, 0.99)** | **0.68 (0.48, 0.98)** |
| >5 | **0.57 (0.34, 0.97)** | **0.54 (0.32, 0.91)** | **0.54 (0.32, 0.91)** |
| **Age, years** | **P= 0.001** | **P= 0.002** | **P= 0.001** |
| <20 | **1 (reference)** | **1 (reference)** | **1 (reference)** |
| 20-24 | **0.97 (0.56, 1.71)** | **0.91 (0.52, 1.60)** | **0.72 (0.39, 1.32)** |
| 25-29 | **1.05 (0.59, 1.87)** | **0.98 (0.55, 1.77)** | **0.73 (0.38, 1.38)** |
| ≥30 | **0.45 (0.24, 0.84)** | **0.44 (0.23, 0.83)** | **0.33 (0.16, 0.66)** |
| **Highest education** | P= 0.08 | P= 0.07 | P= 0.06 |
| No education or incomplete primary | 1 (reference) | 1 (reference) | 1 (reference) |
| Complete primary | 0.71 (0.50, 1.02) | 0.75 (0.52, 1.07) | 0.77 (0.53, 1.10) |
| ≥Secondary | 1.09 (0.67, 1.76) | 1.24 (0.76, 2.02) | 1.31 (0.80, 2.14) |
| **Marital status** | P= 0.42 | P= 0.42 | P= 0.11 |
| Married | 1 (reference) | 1 (reference) | 1 (reference) |
| Separated/divorced/widowed | 1.24 (0.78, 1.96) | 1.04 (0.64, 1.67) | 1.00 (0.62, 1.62) |
| Single | 1.41 (0.84, 2.39) | 1.33 (0.78, 2.26) | 1.53 (0.89, 2.62) |
| **BEHAVIOURAL FACTORS AT ENROLMENT** |  |  |  |
| **AUDIT score [4]** | - | P= 0.38 | P= 0.57 |
| Harmful or hazardous drinking |  | 1 (reference) | 1 (reference) |
| High risk for hazardous drinking |  | 1.22 (0.79, 1.86) | 1.13 (0.74, 1.75) |
| **Age at first sex, years** | - | P= 0.17 | P= 0.28 |
| <16 |  | 1 (reference) | 1 (reference) |
| ≥16 |  | 0.77 (0.54, 1.11) | 0.81 (0.56, 1.18) |
| **Number of lifetime sex partners** | - | **P= 0.03** | **P= 0.04** |
| 0-4 |  | **1 (reference)** | **1 (reference)** |
| 5-9 |  | **1.52 (0.99, 2.36)** | **1.45 (0.94, 2.25)** |
| ≥10 |  | **1.48 (0.93, 2.36)** | **1.39 (0.87, 2.23)** |
| Do not remember |  | **1.91 (1.23, 2.96)** | **1.88 (1.21, 2.92)** |
| **TIME-VARYING BEHAVIOURAL FACTORS** |  |  |  |
| **Number of sex partners in last 3 months** | - | P= 0.59 | P= 0.71 |
| 0 |  | 1 (reference) | 1 (reference) |
| 2 |  | 1.21 (0.77, 1.89) | 1.16 (0.74, 1.82) |
| ≥3 |  | 1.27 (0.71, 2.27) | 1.23 (0.68, 2.20) |
| **Concurrent sex partners in last 3 months** | - | P= 0.30 | P= 0.37 |
| No |  | 1 (reference) | 1 (reference) |
| Yes |  | 1.24 (0.83, 1.87) | 1.21 (0.80, 1.81) |
| **Transactional sex in last 3 months** | - | P= 0.90 | P= 0.96 |
| No |  | 1 (reference) | 1 (reference) |
| Yes |  | 1.03 (0.69, 1.52) | 0.99 (0.67, 1.47) |
| **Contraception** | - | **P= 0.02** | **P= 0.06** |
| None of these |  | **1 (reference)** | **1 (reference)** |
| Condom only |  | **1.50 (1.01, 2.24)** | **1.51 (1.01, 2.25)** |
| Pill (condoms) |  | **1.51 (0.82, 2.79)** | **1.37 (0.74, 2.54)** |
| Injectable (DMPA; condoms) |  | **2.21 (1.39, 3.52)** | **2.00 (1.25, 3.21)** |
| Other hormonal contraceptives |  | **1.76 (0.75, 4.13)** | **1.67 (0.71, 3.93)** |
| **BIOLOGICAL FACTORS AT ENROLMENT** |  |  |  |
| **Ever pregnant** | - | - | **P= 0.02** |
| No |  |  | **1 (reference)** |
| Yes |  |  | **1.96 (1.10, 3.50)** |
| **TIME-VARYING BIOLOGICAL FACTORS** |  |  |  |
| **Current HSV-2 status** | - | - | P= 0.42 |
| Negative |  |  | 1 (reference) |
| Positive (from baseline) |  |  | 0.80 (0.54, 1.18) |
| Positive (seroconverted during follow-up) |  |  | 1.26 (0.45, 3.53) |
| **Current syphilis status** | - | - | P= 0.61 |
| Never infected |  |  | 1 (reference) |
| Previous infection |  |  | 0.73 (0.37, 1.45) |
| Active infection |  |  | 1.09 (0.62, 1.91) |
| **Current Candida status** | - | - | P= 0.20 |
| Negative |  |  | 1 (reference) |
| Positive |  |  | 0.57 (0.22, 1.45) |
| **Current *T. vaginalis* status** | - | - | P= 0.87 |
| Negative |  |  | 1 (reference) |
| Positive |  |  | 0.97 (0.63, 1.48) |
| **Current *N. gonorrhoeae* status** | - | - | **P= 0.01** |
| Negative |  |  | **1 (reference)** |
| Positive |  |  | **2.21 (1.23, 3.97)** |
| **Current vaginal microbiota status** | - | - | P= 0.07 |
| Negative |  |  | 1 (reference) |
| Indeterminate |  |  | 1.70 (1.07, 2.69) |
| Bacteria vaginosis |  |  | 1.34 (0.92, 1.97) |
| **Currently have genital ulcer disease (GUD)** | - | - | P= 0.15 |
| Negative |  |  | 1 (reference) |
| Positive |  |  | 1.71 (0.86, 3.41) |
| **Currently HIV-positive** | - | - | P= 0.51 |
| Negative |  |  | 1 (reference) |
| Positive |  |  | 1.55 (0.45, 5.34) |
| **Currently have vaginal discharge syndrome (VDS) without curd-like discharge** | - | - | P= 0.59 |
| No |  |  | 1 (reference) |
| Yes |  |  | 0.85 (0.48, 1.52) |

[1] Estimated ORs adjusted for visit month, town, age and duration working in facility type (results for these variables shown in bold).
[2] Estimated ORs adjusted for those in [1] and number of lifetime partners and contraception (results for these variables shown in bold).
[3] Estimated ORs adjusted for those in [2] and ever pregnant and gonorrhoea status (results for these variables shown in bold).
[4] Based on responses to ten AUDIT questions. Scores based on responses to each question: 0-7=non-drinker or low-risk,>8 harmful or hazardous drinking.

**Table S2. Final sociodemographic-, behavioural- and biological-level model results for *Neisseria gonorrhoeae* (adjusted odds ratio and 95% CI).**

|  | **Final sociodemographic-level model [1]** | **Final behavioural-level model [2]** | **Final biological-level model [3]** |
| --- | --- | --- | --- |
| **SOCIO-DEMOGRAPHIC CHARACTERISTICS AT ENROLMENT** | | |  |
| **Town** | **P= 0.55** | **P= 0.51** | **P= 0.63** |
| Geita | **1 (reference)** | **1 (reference)** | **1 (reference)** |
| Kahama | **1.34 (0.78, 2.29)** | **1.37 (0.80, 2.35)** | **1.29 (0.76, 2.21)** |
| Shinyanga | **1.07 (0.59, 1.94)** | **1.09 (0.60, 1.97)** | **1.07 (0.59, 1.94)** |
| **Job** | P= 0.42 | P= 0.63 | P= 0.66 |
| Waitress | 1 (reference) | 1 (reference) | 1 (reference) |
| Mamalishe | 0.72 (0.39, 1.34) | 0.82 (0.43, 1.56) | 0.84 (0.44, 1.59) |
| Other | 0.74 (0.43, 1.26) | 0.78 (0.45, 1.34) | 0.78 (0.46, 1.35) |
| **Duration working in facility type, years** | P= 0.90 | P= 0.90 | P= 0.92 |
| ≤1 | 1 (reference) | 1 (reference) | 1 (reference) |
| >1-≤5 | 0.90 (0.53, 1.50) | 0.89 (0.53, 1.49) | 0.90 (0.53, 1.51) |
| >5 | 0.89 (0.43, 1.82) | 0.92 (0.45, 1.89) | 0.92 (0.45, 1.89) |
| **Age, years** | **P<0.001** | **P<0.001** | **P<0.001** |
| <20 | **1 (reference)** | **1 (reference)** | **1 (reference)** |
| 20-24 | **0.62 (0.29, 1.31)** | **0.58 (0.27, 1.23)** | **0.38 (0.17, 0.86)** |
| 25-29 | **0.68 (0.32, 1.45)** | **0.63 (0.30, 1.36)** | **0.37 (0.16, 0.86)** |
| ≥30 | **0.21 (0.09, 0.49)** | **0.21 (0.09, 0.48)** | **0.12 (0.05, 0.31)** |
| **Highest education** | P= 0.26 | P= 0.24 | P= 0.12 |
| No education or incomplete primary | 1 (reference) | 1 (reference) | 1 (reference) |
| Complete primary | 1.43 (0.83, 2.45) | 1.43 (0.83, 2.47) | 1.62 (0.94, 2.79) |
| ≥Secondary | 0.89 (0.39, 2.02) | 0.87 (0.38, 1.99) | 0.93 (0.40, 2.14) |
| **Marital status** | P= 0.06 | P= 0.09 | P= 0.19 |
| Married | 1 (reference) | 1 (reference) | 1 (reference) |
| Separated/divorced/widowed | 1.76 (0.88, 3.53) | 1.63 (0.81, 3.29) | 1.65 (0.83, 3.30) |
| Single | 0.96 (0.43, 2.15) | 0.91 (0.40, 2.07) | 1.09 (0.48, 2.48) |
| **BEHAVIOURAL FACTORS AT ENROLMENT** | |  |  |
| **AUDIT score [4]** | - | **P= 0.04** | **P= 0.09** |
| Non-drinker or low risk drinking |  | **1 (reference)** | **1 (reference)** |
| Harmful or hazardous drinking |  | **1.83 (1.03, 3.25)** | **1.66 (0.94, 2.94)** |
| **Age at first sex, years** | - | P= 0.61 | P= 0.91 |
| <16 |  | 1 (reference) | 1 (reference) |
| ≥16 |  | 0.87 (0.52, 1.46) | 0.97 (0.58, 1.63) |
| **Number of lifetime sex partners** | - | P= 0.89 | P= 0.86 |
| 0-4 |  | 1 (reference) | 1 (reference) |
| 5-9 |  | 1.13 (0.61, 2.08) | 1.02 (0.55, 1.88) |
| ≥10 |  | 0.93 (0.47, 1.85) | 0.84 (0.43, 1.67) |
| Do not remember |  | 0.84 (0.43, 1.67) | 0.78 (0.39, 1.55) |
| **TIME-VARYING BEHAVIOURAL FACTORS** | |  |  |
| **Number of sex partners in last 3 months** | - | P= 0.41 | P= 0.54 |
| 0 |  | 1 (reference) | 1 (reference) |
| 2 |  | 1.50 (0.84, 2.69) | 1.40 (0.78, 2.52) |
| ≥3 |  | 1.18 (0.50, 2.78) | 1.10 (0.46, 2.59) |
| **Concurrent sex partners in last 3 months** | - | P= 0.33 | P= 0.48 |
| No |  | 1 (reference) | 1 (reference) |
| Yes |  | 1.33 (0.76, 2.35) | 1.23 (0.70, 2.18) |
| **Transactional sex in last 3 months** | - | P= 0.11 | P= 0.15 |
| No |  | 1 (reference) | 1 (reference) |
| Yes |  | 1.55 (0.91, 2.63) | 1.48 (0.87, 2.51) |
| **Contraception** | - | P= 0.37 | P= 0.48 |
| None of these |  | 1 (reference) | 1 (reference) |
| Condom only |  | 1.18 (0.69, 2.02) | 1.18 (0.69, 2.02) |
| Pill (+/-condoms) |  | 1.27 (0.54, 3.01) | 1.07 (0.45, 2.53) |
| Injectable (DMPA; +/-condoms) |  | 1.66 (0.86, 3.21) | 1.37 (0.71, 2.66) |
| Other hormonal contraceptives |  | 0.34 (0.04, 2.75) | 0.29 (0.04, 2.32) |
| **BIOLOGICAL FACTORS AT ENROLMENT** | |  |  |
| **Ever pregnant** | - | - | **P= 0.003** |
| No |  |  | **1 (reference)** |
| Yes |  |  | **3.39 (1.41, 8.16)** |
| **TIME-VARYING BIOLOGICAL FACTORS** | |  |  |
| **Current HSV-2 status** | - | - | P= 0.27 |
| Negative |  |  | 1 (reference) |
| Positive (from baseline) |  |  | 1.43 (0.78, 2.63) |
| Positive (seroconverted during follow-up) |  |  | 2.59 (0.73, 9.19) |
| **Current syphilis status** | - | - | P= 0.47 |
| Never infected |  |  | 1 (reference) |
| Previous infection |  |  | 0.54 (0.18, 1.59) |
| Active infection |  |  | 0.85 (0.36, 2.01) |
| **Current Candida status** | - | - | P= 0.70 |
| Negative |  |  | 1 (reference) |
| Positive |  |  | 1.22 (0.45, 3.30) |
| **Current *T. vaginalis* status** | - | - | P= 0.34 |
| Negative |  |  | 1 (reference) |
| Positive |  |  | 1.33 (0.75, 2.36) |
| **Current *C. trachomatis* status** | - | - | **P= 0.02** |
| Negative |  |  | **1 (reference)** |
| Positive |  |  | **2.19 (1.19, 4.05)** |
| **Current vaginal microbiota status** | - | - | P= 0.38 |
| Negative |  |  | 1 (reference) |
| Indeterminate |  |  | 1.38 (0.71, 2.68) |
| Bacteria vaginosis |  |  | 1.44 (0.84, 2.45) |
| **Currently have genital ulcer disease (GUD)** | - | - | P= 0.22 |
| Negative |  |  | 1 (reference) |
| Positive |  |  | 1.83 (0.73, 4.62) |
| **Currently have vaginal discharge syndrome (VDS) without curd-like discharge** | - | - | P= 0.31 |
| No |  |  | 1 (reference) |
| Yes |  |  | 1.47 (0.71, 3.03) |

[1] Estimated ORs adjusted for visit month, town and age (results for these variables shown in bold).
[2] Estimated ORs adjusted for those in [1] and AUDIT (results for these variables shown in bold).
[3] Estimated ORs adjusted for those in [2] and ever pregnant and chlamydia status (results for these variables shown in bold).
[4] Based on responses to ten AUDIT questions. Scores based on responses to each question: 0-7=non-drinker or low-risk,>8 harmful or hazardous drinking.

**Table S3. Final sociodemographic-, behavioural- and biological-level model results for *Trichomonas vaginalis* (adjusted odds ratio and 95% CI).**

|  | **Final sociodemographic-level model [1]** | **Final behavioural-level model [2]** | **Final biological-level model [3]** |
| --- | --- | --- | --- |
| **SOCIO-DEMOGRAPHIC CHARACTERISTICS AT ENROLMENT** | |  |  |
| **Town** | **P= 0.75** | **P= 0.75** | **P= 0.88** |
| Geita | **1 (reference)** | **1 (reference)** | **1 (reference)** |
| Kahama | **1.15 (0.76, 1.75)** | **1.15 (0.76, 1.75)** | **1.11 (0.73, 1.67)** |
| Shinyanga | **1.00 (0.65, 1.54)** | **1.00 (0.65, 1.54)** | **1.07 (0.70, 1.64)** |
| **Job** | P= 0.38 | P= 0.38 | P= 0.53 |
| Waitress | 1 (reference) | 1 (reference) | 1 (reference) |
| Mamalishe | 0.73 (0.45, 1.18) | 0.73 (0.45, 1.18) | 0.78 (0.48, 1.25) |
| Other | 0.82 (0.54, 1.23) | 0.82 (0.54, 1.23) | 0.85 (0.57, 1.27) |
| **Duration working in facility type, years** | P= 0.24 | P= 0.24 | P= 0.23 |
| ≤1 | 1 (reference) | 1 (reference) | 1 (reference) |
| >1-≤5 | 0.89 (0.59, 1.33) | 0.89 (0.59, 1.33) | 0.86 (0.57, 1.28) |
| >5 | 0.64 (0.38, 1.08) | 0.64 (0.38, 1.08) | 0.64 (0.38, 1.07) |
| **Age, years** | **P= 0.82** | **P= 0.82** | **P= 0.84** |
| <20 | **1 (reference)** | **1 (reference)** | **1 (reference)** |
| 20-24 | **1.27 (0.63, 2.55)** | **1.27 (0.63, 2.55)** | **1.35 (0.68, 2.69)** |
| 25-29 | **1.15 (0.55, 2.43)** | **1.15 (0.55, 2.43)** | **1.23 (0.59, 2.56)** |
| ≥30 | **1.04 (0.49, 2.22)** | **1.04 (0.49, 2.22)** | **1.20 (0.57, 2.53)** |
| **Highest education** | **P= 0.001** | **P= 0.001** | **P= 0.003** |
| No education or incomplete primary | **1 (reference)** | **1 (reference)** | **1 (reference)** |
| Complete primary | **0.52 (0.36, 0.77)** | **0.52 (0.36, 0.77)** | **0.55 (0.38, 0.81)** |
| ≥Secondary | **0.40 (0.22, 0.74)** | **0.40 (0.22, 0.74)** | **0.44 (0.24, 0.80)** |
| **Marital status** | **P= 0.001** | **P= 0.001** | **P= 0.003** |
| Married | **1 (reference)** | **1 (reference)** | **1 (reference)** |
| Separated/divorced/widowed | **2.42 (1.49, 3.93)** | **2.42 (1.49, 3.93)** | **2.27 (1.41, 3.66)** |
| Single | **1.96 (1.08, 3.57)** | **1.96 (1.08, 3.57)** | **1.95 (1.08, 3.51)** |
| **BEHAVIOURAL FACTORS AT ENROLMENT** | |  |  |
| **AUDIT score [4]** | - | P= 0.79 | P= 0.49 |
| Non-drinker or low risk drinking |  | 1 (reference) | 1 (reference) |
| Harmful or hazardous drinking |  | 0.94 (0.57, 1.53) | 0.84 (0.52, 1.37) |
| **Age at first sex, years** | - | P= 0.06 | P= 0.10 |
| <16 |  | 1 (reference) | 1 (reference) |
| ≥16 |  | 0.69 (0.47, 1.02) | 0.73 (0.50, 1.06) |
| **Number of lifetime sex partners** | - | P= 0.95 | P= 0.96 |
| 0-4 |  | 1 (reference) | 1 (reference) |
| 5-9 |  | 1.01 (0.63, 1.62) | 1.01 (0.63, 1.61) |
| ≥10 |  | 0.92 (0.55, 1.53) | 0.91 (0.55, 1.50) |
| Do not remember |  | 1.09 (0.67, 1.78) | 1.04 (0.65, 1.68) |
| **TIME-VARYING BEHAVIOURAL FACTORS** | |  |  |
| **Number of sex partners in last 3 months** | - | P= 0.78 | P= 0.95 |
| 0 |  | 1 (reference) | 1 (reference) |
| 2 |  | 0.99 (0.65, 1.52) | 0.98 (0.64, 1.50) |
| ≥3 |  | 1.22 (0.69, 2.15) | 1.08 (0.61, 1.91) |
| **Concurrent sex partners in last 3 months** | - | P= 0.98 | P= 0.81 |
| No |  | 1 (reference) | 1 (reference) |
| Yes |  | 0.99 (0.67, 1.48) | 0.95 (0.64, 1.41) |
| **Transactional sex in last 3 months** | - | P= 0.20 | P= 0.34 |
| No |  | 1 (reference) | 1 (reference) |
| Yes |  | 1.26 (0.88, 1.81) | 1.19 (0.83, 1.71) |
| **Contraception** | - | P= 0.53 | P= 0.53 |
| None of these |  | 1 (reference) | 1 (reference) |
| Condom only |  | 1.02 (0.72, 1.46) | 1.02 (0.72, 1.46) |
| Pill (+/-condoms) |  | 0.99 (0.54, 1.80) | 1.13 (0.62, 2.05) |
| Injectable (DMPA; +/-condoms) |  | 0.69 (0.41, 1.14) | 0.71 (0.43, 1.18) |
| Other hormonal contraceptives |  | 1.37 (0.55, 3.42) | 1.44 (0.58, 3.57) |
| **BIOLOGICAL FACTORS AT ENROLMENT** | |  |  |
| **Ever pregnant** | - | - | P= 0.69 |
| No |  |  | 1 (reference) |
| Yes |  |  | 0.89 (0.51, 1.57) |
| **TIME-VARYING BIOLOGICAL FACTORS** | |  |  |
| **Current HSV-2 status** | - | - | P= 0.46 |
| Negative |  |  | 1 (reference) |
| Positive (from baseline) |  |  | 1.29 (0.82, 2.03) |
| Positive (seroconverted during follow-up) |  |  | 0.84 (0.28, 2.53) |
| **Current syphilis status** | - | - | P= 0.08 |
| Never infected |  |  | 1 (reference) |
| Previous infection |  |  | 1.01 (0.58, 1.77) |
| Active infection |  |  | 1.85 (1.08, 3.17) |
| **Current Candida status** | - | - | P= 0.73 |
| Negative |  |  | 1 (reference) |
| Positive |  |  | 1.13 (0.57, 2.22) |
| **Current *C. trachomatis* status** | - | - | P= 0.86 |
| Negative |  |  | 1 (reference) |
| Positive |  |  | 1.05 (0.63, 1.75) |
| **Current *N. gonorrhoeae* status** | - | - | P= 0.62 |
| Negative |  |  | 1 (reference) |
| Positive |  |  | 1.20 (0.59, 2.44) |
| **Current vaginal microbiota status** | - | - | **P<0.001** |
| Negative |  |  | **1 (reference)** |
| Indeterminate |  |  | **4.37 (2.84, 6.72)** |
| Bacteria vaginosis |  |  | **2.28 (1.58, 3.30)** |
| **Currently have genital ulcer disease (GUD)** | - | - | P= 0.73 |
| Negative |  |  | 1 (reference) |
| Positive |  |  | 0.86 (0.37, 1.99) |
| **Currently HIV-positive** | - | - | P= 0.82 |
| Negative |  |  | 1 (reference) |
| Positive |  |  | 0.86 (0.25, 3.00) |
| **Currently have vaginal discharge syndrome (VDS) without curd-like discharge** | - | - | P= 0.13 |
| No |  |  | 1 (reference) |
| Yes |  |  | 1.49 (0.90, 2.47) |

[1] Estimated ORs adjusted for visit month, town, age, education and marital status (results for these variables shown in bold).
[2] Estimated ORs adjusted for those in [1] (same as [1] since no behavioural factors included; results for these variables shown in bold).
[3] Estimated ORs adjusted for those in [2] and current vaginal microbiota assessed by Nugent score (results for these variables shown in bold).
[4] Based on responses to ten AUDIT questions. Scores based on responses to each question: 0-7=non-drinker or low-risk,>8 harmful or hazardous drinking.

**Table S4. Final sociodemographic-, behavioural- and biological-level model results for active syphilis (high titre) [1] (adjusted odds ratio and 95% CI).**

|  | **Final sociodemographic-level model [2]** | **Final behavioural-level model [3]** | **Final biological-level model [4]** |
| --- | --- | --- | --- |
| **SOCIO-DEMOGRAPHIC CHARACTERISTICS** | |  |  |
| **Town** | **P=0.19** | **P=0.52** | **P=0.57** |
| Geita | **1 (reference)** | **1 (reference)** | **1 (reference)** |
| Kahama | **0.76 (0.38,1.54)** | **0.80 (0.37,1.75)** | **0.84 (0.38,1.87)** |
| Shinyanga | **0.48 (0.21,1.10)** | **0.61 (0.26,1.46)** | **0.63 (0.26,1.51)** |
| **Job** | P=0.38 | P=0.66 | P=0.62 |
| Waitress | 1 (reference) | 1 (reference) | 1 (reference) |
| Mamalishe | 0.65 (0.28,1.50) | 0.92 (0.38,2.24) | 1.03 (0.42,2.56) |
| Other | 0.64 (0.31,1.31) | 0.69 (0.30,1.58) | 0.69 (0.30,1.60) |
| **Duration working in facility type, years** | P=0.18 | P=0.32 | P=0.36 |
| ≤1 | 1 (reference) | 1 (reference) | 1 (reference) |
| >1-≤5 | 1.29 (0.66,2.50) | 1.32 (0.63,2.75) | 1.41 (0.67,2.96) |
| >5 | 0.50 (0.17,1.48) | 0.60 (0.20,1.82) | 0.67 (0.22,2.06) |
| **Age, years** | **P=0.16** | **P=0.33** | **P=0.33** |
| <20 | **1 (reference)** | **1 (reference)** | **1 (reference)** |
| 20-24 | **1.18 (0.42,3.32)** | **1.33 (0.41,4.32)** | **1.36 (0.41,4.53)** |
| 25-29 | **0.46 (0.13,1.57)** | **0.63 (0.17,2.38)** | **0.68 (0.18,2.62)** |
| ≥30 | **1.16 (0.41,3.32)** | **1.45 (0.45,4.70)** | **1.60 (0.48,5.34)** |
| **Highest education** | **P<0.001** | **P=0.001** | **P=0.004** |
| No education or incomplete primary | **1 (reference)** | **1 (reference)** | **1 (reference)** |
| Complete primary or ≥Secondary | **0.31 (0.17,0.57)** | **0.32 (0.16,0.63)** | **0.36 (0.18,0.72)** |
| **Marital status** | P=0.98 | P=0.91 | P=0.93 |
| Married | 1 (reference) | 1 (reference) | 1 (reference) |
| Separated/divorced/widowed | 1.09 (0.49,2.44) | 0.98 (0.41,2.34) | 0.90 (0.37,2.20) |
| Single | 1.10 (0.40,2.98) | 1.18 (0.40,3.49) | 1.06 (0.35,3.23) |
| **BEHAVIOURAL FACTORS** |  |  |  |
| **AUDIT score [5]** | - | P=0.59 | P=0.76 |
| Harmful or hazardous drinking |  | 1 (reference) | 1 (reference) |
| High risk for hazardous drinking |  | 0.76 (0.28,2.11) | 0.85 (0.31,2.37) |
| **Age at first sex, years** | - | P=0.36 | P=0.33 |
| <16 |  | 1 (reference) | 1 (reference) |
| ≥16 |  | 0.71 (0.34,1.48) | 0.69 (0.33,1.45) |
| **Number of lifetime sex partners** | - | P=0.50 | P=0.34 |
| 0-4 |  | 1 (reference) | 1 (reference) |
| 5-9 |  | 0.72 (0.28,1.87) | 0.67 (0.25,1.78) |
| ≥10 |  | 0.47 (0.15,1.50) | 0.42 (0.13,1.38) |
| Do not remember |  | 0.99 (0.39,2.49) | 1.07 (0.42,2.71) |
| **Number of sex partners in last 3 months** | - | P=0.28 | P=0.41 |
| 0 |  | 1 (reference) | 1 (reference) |
| 2 |  | 0.14 (0.01,1.97) | 0.23 (0.01,3.78) |
| ≥3 |  | 0.08 (0.00,1.41) | 0.14 (0.01,2.65) |
| **Concurrent sex partners in last 3 months** | - | **P=0.004** | **P=0.005** |
| No |  | **1 (reference)** | **1 (reference)** |
| Yes |  | **2.71 (1.39,5.28)** | **2.68 (1.36,5.27)** |
| **Transactional sex in last 3 months** | - | P=0.97 | P=0.90 |
| No |  | 1 (reference) | 1 (reference) |
| Yes |  | 1.01 (0.48,2.12) | 0.95 (0.45,2.02) |
| **Contraception** | - | P=0.89 | P=0.92 |
| None of these |  | 1 (reference) | 1 (reference) |
| Condom only |  | 0.76 (0.35,1.65) | 0.72 (0.33,1.60) |
| Pill (condoms) |  | 0.90 (0.25,3.23) | 0.91 (0.25,3.33) |
| Injectable (DMPA; condoms) |  | 0.57 (0.18,1.79) | 0.66 (0.21,2.08) |
| Other hormonal contraceptives |  | 0.90 (0.11,7.36) | 1.00 (0.12,8.24) |
| **BIOLOGICAL FACTORS** |  |  |  |
| **Ever pregnant** | - | - | P=0.59 |
| No |  |  | 1 (reference) |
| Yes |  |  | 0.76 (0.28,2.03) |
| **Current HSV-2 status** | - | - | P=0.69 |
| Negative |  |  | 1 (reference) |
| Positive (from baseline or seroconverted during follow-up) |  |  | 0.84 (0.37,1.95) |
| **Current Candida status** | - | - | P=0.29 |
| Negative |  |  | 1 (reference) |
| Positive |  |  | 2.13 (0.58,7.83) |
| **Current *T. vaginalis* status** | - | - | **P=0.001** |
| Negative |  |  | **1 (reference)** |
| Positive |  |  | **3.27 (1.66,6.43)** |
| **Current *C. trachomatis* status** | - | - | P=0.73 |
| Negative |  |  | 1 (reference) |
| Positive |  |  | 0.83 (0.27,2.50) |
| **Current *N. gonorrhoeae* status** | - | - | P=0.21 |
| Negative |  |  | 1 (reference) |
| Positive |  |  | 2.51 (0.68,9.28) |
| **Current vaginal microbiota status** | - | - | P=0.08 |
| Negative |  |  | 1 (reference) |
| Indeterminate |  |  | 0.62 (0.19,1.97) |
| Bacterial vaginosis |  |  | 1.72 (0.78,3.80) |
| **Currently have genital ulcer disease (GUD)** | - | - | P=0.92 |
| Negative |  |  | 1 (reference) |
| Positive |  |  | 1.08 (0.22,5.20) |
| **Currently have vaginal discharge syndrome (VDS) without curd-like discharge** | - | - | P=0.76 |
| No |  |  | 1 (reference) |
| Yes |  |  | 0.83 (0.26,2.70) |

[1] Syphilis model used data only from the enrolment visit since there were very few incident infections.

[2] Estimated ORs adjusted for town, age and education (results for these variables shown in bold).
[3] Estimated ORs adjusted for those in [1] and concurrent partners in last 3 months (results for these variables shown in bold).
[4] Estimated ORs adjusted for those in [2] and current trichomoniasis status (results for these variables shown in bold).
[5] Based on responses to ten AUDIT questions. Scores based on responses to each question: 0-7=non-drinker or low-risk,>8 harmful or hazardous drinking.
